# Immunotherapy of Neuromyelitis Optica

**DOI:** 10.1155/2013/741490

**Published:** 2013-12-25

**Authors:** Benjamin Bienia, Roumen Balabanov

**Affiliations:** Department of Neurological Sciences, Multiple Sclerosis Center, Rush University Medical Center, 1725 W. Harrison Street, Suite 309, Chicago, IL 60612, USA

## Abstract

Neuromyelitis optica (NMO) is a chronic inflammatory disease of the central nervous system that affects the optic nerves and spinal cord resulting in visual impairment and myelopathy. There is a growing body of evidence that immunotherapeutic agents targeting T and B cell functions, as well as active elimination of proinflammatory molecules from the peripheral blood circulation, can attenuate disease progression. In this review, we discuss the immunotherapeutic options and the treatment strategies in NMO. We also analyze the pathogenic mechanisms of the disease in order to provide recommendations regarding treatments.

## 1. Introduction

Neuromyelitis optica (NMO), also known as Devic's disease, is a chronic inflammatory disease of the central nervous system (CNS) that preferentially targets the optic nerves and spinal cord [1]. The overall disease incidence has been estimated at 1 : 100,000 and that it has a predilection for middle-aged, non-Caucasian females [2]. NMO spectrum disorders (NMOSD) encompass a variation of this classical picture in that patients may have brain involvement or a more limited presentation such as isolated transverse myelitis or an optic neuritis [3]. Historically, many thought of NMO as a rare variant of multiple sclerosis (MS). Given the identification of unique clinical and radiological differences and the discovery of the NMO-IgG, an autoantibody against aquaporin-4 (aqp4), it is now understood to be its own entity with distinct pathogenesis, diagnostic criteria, prognosis, and treatment [1–5].

Until recently, NMO was considered a disease of limited therapeutic options and poor prognosis. Research over the last decade brought new understanding of the disease pathogenesis that translated into immunotherapy directed against this disease. Moreover, there is a growing body of evidence that NMO can be controlled by immunotherapeutics targeting its cellular and humoral immune mechanisms. We review the immunotherapy of NMO, the various treatment options, and the clinical strategies that are typically encountered in practice.

## 2. Neuromyelitis Optica: An Overview 

NMO is a neurological disorder that classically presents as a case of severe bilateral optic neuritis associated with a transverse myelitis [1–5]. The typical disease onset is either acute or subacute, and the symptoms are likely to persist without treatment. Optic neuritis results in decreased or a complete loss of vision. Transverse myelitis is usually extensive and spans more than 3 consecutive vertebral segments. Deficits related to myelitis include paralysis and sensory loss below the lesion level along with gait impairment. Additional complications may include phrenic nerve paralysis, loss of sphincter control, dysautonomia, and painful tonic spasms. Brainstem (medulla oblongata and area postrema) can be involved at times with resultant persistent nausea and hiccups [6].

Magnetic resonance imaging (MRI) is used for diagnosis and monitoring of the disease [1, 4]. Optic nerve and spinal cord lesions appear as hyperintense on T2- and hypointense on T1-weighted images and enhance with gadolinium when they are inflamed. In the acute phase, the inflamed lesions also enlarge in size secondary to tissue edema. Inflammation may persist for months and result in tissue atrophy [7]. MRI lesions involving the brainstem, hypothalamus, and periventricular white matter may be seen in typical NMOSD and sometimes late in the disease course of NMO [1–3]. Independent of imaging, visual evoked potentials and CSF studies can be helpful in establishing the diagnosis [4, 8]. Optic coherence tomography (OCT) may also be used to monitor the extent and the degree of progression of optic neuropathy [9].

NMO follows a relapsing-remitting clinical course in 70–90% of all patients [2]. Such a clinical course is correlated with female gender, older age of disease onset, longer (>3 months) optic neuritis-myelitis interval, and presence of systemic autoimmunity [2, 3]. Seropositivity for anti-aqp4 antibody is also a strong predictor for future disease relapses [10]. A monophasic clinical course tends to occur in young males. Neurological disability in relapsing-remitting disease appears to be a cumulative result of disease relapses [2]. After five years, approximately 50% of affected individuals have significant visual or motor impairment and require assistive devices for ambulation [2]. This time frame of five years is also notable for a mortality rate of 32% with the relapsing-remitting disease and 10% with the monophasic disease. Most patients expire from disease complications such as respiratory failure, urosepsis, and pulmonary embolism [2–4].

The etiology of NMO is unknown but it is believed to be an autoimmune disorder triggered by an environmental factor, possibly an infection, in genetically susceptible individuals [11–13]. The principal effector in NMO is the self-reactive, complement-activating anti-aqp4 antibody [14]. Aqp4 is a transmembrane protein that regulates the flow of water in cells. It is expressed by CNS astrocytes and astrocytic processes surrounding small blood vessels at the glia limitans [15]. The autoantibody has the capacity to bind to aqp4 on the astrocytic foot processes and then recruit and activate complement. This leads to the mobilization of polymorphonuclear cells (neutrophils and eosinophils), inflammation, and tissue swelling [16, 17].

Recent studies indicate that Th17 cells (a T cell subset producing interleukin 17) specific to aqp4 may also be involved in the disease pathogenesis [18]. They are implicated in the breakdown of the blood-brain barrier allowing extravasation of anti-aqp4 antibody and complement, along with recruitment of polymorphonuclear cells to the lesion sites. Pathologically, NMO lesions involve both the white and gray matter. They contain perivascular deposits of immune complexes, activated complement, and inflammatory cellular infiltrates [19]. The cellular infiltrates are composed of mononuclear and polymorphonuclear cells. Astrocytes targeted by the autoimmune response display cytopathic changes and downregulate the expression of aqp4 in a vasculocentric pattern [20]. Vascular hyalinization, tissue necrosis, demyelination, and gliosis commonly accompany the inflammatory process [18–22].

## 3. Immunotherapy of NMO

**Figure 1:**
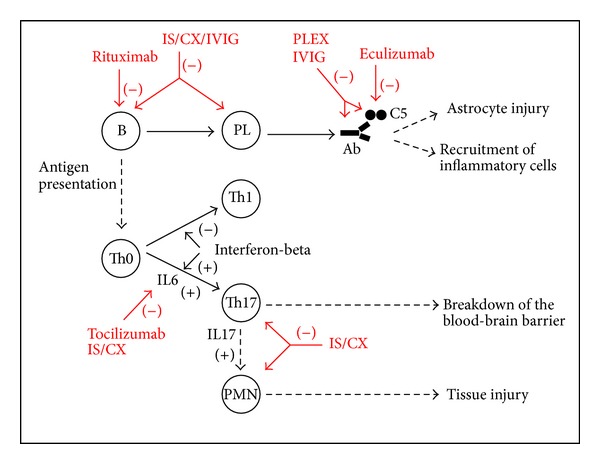
Summary of the mechanisms of action of immunotherapies in NMO. Abbreviations: B = B cell, C5 = protein 5 of complement, CX = cytotoxic agent, IL = interleukin, IS = immunosuppressant, IVIG = intravenous immunoglobulin, PL = plasma  cell, PMN = polymorphonuclear cell, Th = T helper cell.

Immunotherapy of NMO is based on the current understanding of its pathogenesis. As summarized above, lesion formation involves interplay between cellular and humoral immune responses. It appears that the autoimmune reaction arises in the periphery with the appearance of anti-aqp4 antibodies and Th17 cells; then it progresses in cascade-like fashion. There are several points of augmentation and diversification of the autoimmune reaction, including complement activation and release of interleukin 17, which have proinflammatory and chemotactic effects. This contributes to the recruitment of mononuclear and polymorphonuclear cells to the sites of initial inflammation. As the inflammatory reaction unfolds, a number of nonspecific injurious mechanisms become involved including vascular damage, tissue swelling, oxidative stress, astrocyte injury, and secondary demyelination. These processes can be suppressed by using immunotherapeutic agents targeting T and B cells (immunosuppressant, cytotoxic, and biologic agents) or by actively removing the pro-inflammatory factors from the peripheral blood circulation (therapeutic plasma exchange) (Figure 1). These therapeutic approaches are nonspecific to the self-reactive cells or antibodies but affect the immune system globally. Inflammation in NMO is necrotizing in nature and cannot be reversed; it can only be prevented or minimized with effective treatment.

Immunotherapy of NMO is divided into two parts: rescue therapy of an acute disease relapse and disease-modifying therapy. The goal of rescue therapy is to suppress the acute inflammatory process in order to achieve functional recovery in patients. Early and effective rescue therapy is essential in minimizing the degree of permanent tissue damage and neurological disability. Corticosteroids and plasma exchange (PLEX) are the most commonly used therapeutic modalities in acute settings. Corticosteroids exert global immunosuppressive and anti-inflammatory effects, whereas PLEX removes antibodies, complement, and cytokines from the blood. The effects of both treatment modalities are rapid and can be appreciated within days of their initiation. Corticosteroids are administered intravenously. The usual treatment regimen is that of methylprednisolone 1000 mg daily for 5 days, followed by an extended oral prednisone taper starting at 60–100 mg per day [23]. If the disease is refractory to corticosteroids, PLEX therapy should be considered. PLEX can be beneficial to patients with acute NMO and is frequently recommended as a second line therapy in refractory cases [24, 25]. In practice, methylprednisolone is administered first and if there is no treatment response within three to four days, PLEX may be initiated. PLEX is administered every other day (1.5x plasma volume per each exchange) over the course of two weeks. In our clinical experience, most patients exhibited functional improvement after four to six PLEX treatments. The patient's response to initial rescue therapy may not be immediate and should be reevaluated within a few weeks after its completion. In cases of a poor response or an early disease relapse, one may consider repeating the corticosteroid/PLEX treatment or using cytotoxic agents such as cyclophosphamide. The latter is administered as several monthly infusions at 0.5–1 g/m^2^ and can be beneficial in refractory cases, particularly in patients with concomitant systemic autoimmune diseases [26].

The goal of disease-modifying treatment is to maintain disease remission and prevent future relapses. It is important to keep in mind that the majority of NMO patients are likely to have a relapsing-remitting disease. As such, their neurological disability will be cumulative and related to the frequency and severity of their disease relapses [27]. As many as 60% of all patients are likely to develop a disease relapse in the first year and 95% within three years of diagnosis [2]. In this respect early recognition of the predictors of relapsing-remitting disease is important. There are no randomized double blind placebo-controlled studies that have demonstrated the efficacy of any of the aforementioned treatment options. Most of the current knowledge is derived from anecdotal or small retrospective studies. Therefore, recommended treatments are based on the current understanding of disease pathogenesis, observed responses to treatment, tolerability, and the availability of resources.

Immunosuppressant agents interfering with the function of T and B cells have been shown to prevent disease relapses and reduce neurological disability in NMO. They can be viewed as steroid-sparing agents extending the beneficial effect of the rescue corticosteroid therapy. Azathioprine, perhaps the most commonly used oral immunosuppressant agent in NMO, primarily suppresses T cell function [23, 28]. The largest retrospective study involving 99 patients reported that azathioprine decreased the annualized relapse rate by 76% and either improved or stabilized disability in 40% of patients in a twelve-month period [28]. Azathioprine may be initiated at a dose of 50 mg daily or less and subsequently increased as tolerated to a target dose of 2-3 mg/kg/day (approximately 200–300 mg daily) either during or immediately after the intravenous methylprednisolone treatment. Doses lower than 2 mg/kg/day may have a limited effect on disease activity [28]. Prednisone in a prolonged tapering regimen from a dose of 100 mg down to 10 mg over a year may be added in order to compensate for the slow mechanism of action of azathioprine and to broaden the spectrum of immunosuppression. Once disease remission is achieved, then the medication can be continued as monotherapy for years at the lowest effective dose [23, 28].

Mycophenolate mofetil is another oral immunosuppressant that has the advantage of suppressing the proliferation of both T and B cells, as well as the production of antibodies by plasma cells [29]. It is effective in patients with NMO at an average dose of 2000 mg daily and is generally well tolerated [29]. In a retrospective study involving 25 patients, treatment with mycophenolate mofetil was reported to decrease the annualized relapse rate in 71% of patients and improves disability in 91% of patients over a median follow-up of twenty-eight months [29]. Similar to azathioprine, adding corticosteroids (intravenous or oral) to mycophenolate mofetil treatment can potentiate its efficacy, particularly in the first several months of treatment. In addition, prednisone can prevent disease relapses in some cases as a sole disease-modifying agent. In these instances, doses at 25 mg or above every other day are necessary to maintain disease remission [30].

A few small case studies reported the disease-modifying effect of intermittent PLEX on NMO relapses [31, 32]. This approach may be used as a long-term extension of the rescue PLEX, especially in patients who have had a dramatic initial treatment response. It can be also considered as an alternative to immunosuppressants in the setting of treatment failure or significant side effects. While the frequency of intermittent PLEX sessions should be established empirically based on the duration of treatment-induced disease remissions, it is usually administered once every two to three months [31]. More frequent regimens on a weekly basis can be considered as well [32]. As previously noted, a small dose of daily prednisone of 5–20 mg daily can provide an add-on therapeutic benefit [31].

Rituximab is a monoclonal antibody against B cells (anti-CD 20), which can directly deplete this cell population from the peripheral blood circulation in a matter of a few weeks. This effect is global as B cells serve as precursors of antibody-producing plasma cells and are involved in the processes of antigen presentation and T cell activation. Rituximab has been reported to be effective, particularly in patients who have failed oral immunosuppressant therapy [33–35]. The two largest retrospective studies, which included more than 20 patients, each reported significant reduction in the annualized relapse rate and improvement in neurological disability in more than 80%–90% of cases in nearly a two-year period [34, 35]. The drug can be administered intravenously at 375 mg/m once weekly for four weeks or at a flat dose of 1 g two weeks apart. Periodic retreatments are often necessary depending on the clinical response [36, 37]. Notably, the rituximab dose and frequency of administration can be tailored to the level of peripheral B cells, which should be maintained at zero.

Eculizumab is another monoclonal antibody that neutralizes complement protein 5 (anti-C5), thereby inhibiting the propagation of the complement cascade, the recruitment of inflammatory cells, and the formation of the membrane-attack complex. Recently, a small open-label study involving 14 NMO-IgG seropositive patients reported that biweekly intravenous administration of 900 mg of eculizumab (after a titration period) had a significant impact on the disease [38]. Twelve of 14 patients became relapse free after twelve months of treatment. Significant improvements in visual acuity and median disability scores were reported as well. None of the patients developed disease worsening. However, a return of disease activity was observed in 5 patients following discontinuation of the medication. Eculizumab administration was associated with significant suppression of serum complement activity and reduction of C5 levels in CSF, whereas medication discontinuation was associated with their normalization. NMO-IgG titers measured throughout the study remained unchanged.

Other authors reported benefit with other agents including cyclosporine, mitoxantrone, methotrexate, intravenous immunoglobulin (IVIG), and tocilizumab (anti-interleukin 6) [39–43]. For instance, intermittent administration of IVIG may be useful as an acute and chronic treatment in patients who have failed standard immunosuppressive therapy [42]. Tocilizumab blockade of interleukin 6, a cytokine that potentiates B cell survival and Th17 immune responses, may be effective in patients with highly active disease that are unresponsive to multiple immunosuppressive and cell depleting therapies [43]. Overall, these studies are retrospective, anecdotal, or small in size [39–43]. Nonetheless, NMO is a rare autoimmune disease that can be refractory to multiple treatments and every positive experience can be of potential value in clinical practice.

## 4. Additional Considerations

Currently, there is no biomarker for therapeutic response in NMO. There are observations correlating effective immunotherapy to a decrease in anti-aqp4 antibody levels of patients [44]. However, there are no definitive studies establishing the significance of this autoantibody as a biomarker of treatment response. Moreover, it appears that seropositive and seronegative NMO patients do not differ in their responses to immunotherapy [45, 46]. Most of the treatment assessments are based on general neurologic or empirical principles. These include change in relapse rate or neurological disability and appearance of new or gadolinium-enhancing lesions on MRI. In a few studies neurological improvement in patients treated with PLEX was reported to be associated with early treatment, rapid initial response, male gender, preserved leg reflexes, and absence of atrophy on MRI [46, 47]. In addition, there is evidence that preservation of retinal nerve fiber layer on OCT can be associated with a good treatment response to corticosteroids and PLEX [9, 32].

Certain laboratory tests reflecting the mechanism of action of medications may be useful in monitoring and predicting treatment responses in patients. For instance, a slight elevation of the erythrocyte mean corpuscular volume (MCV) more than 5 points above baseline following treatment with azathioprine may correlate with effective immune suppression and associated decline in patients' annualized relapse rate [28]. This increase in size of red blood cells is a metabolic effect of the medication and in fact some of its metabolites can be directly measured in these cells [48]. Elevation of MCV is well tolerated and is reversible with discontinuation of azathioprine [48]. Levels of mycophenolate mofetil metabolites such as mycophenolic acid and mycophenolic acid glucuronide can be directly measured in patient's blood [49]. Even though there are studies indicating the significance of therapeutic monitoring of mycophenolic acid in organ transplantation, its value in NMO remains to be established.

Rituximab is a cell depleting monoclonal antibody whose clinical benefit negatively correlates with levels of peripheral B cells [36, 50]. In some reported cases, rituximab failure was associated with rapid recovery of B cells after treatment. However, disease worsening on rituximab may have a more complex nature. Initial response to corticosteroids was identified as a negative predictor in some patients. This was hypothesized as being due to predominant T cell involvement with relatively less B cell involvement in the disease's pathogenesis [50]. Early disease worsening can be also due to extensive B cell death and secondary nonspecific activation of the immune system or by transient elevation of the anti-aqp4 antibody [50–52]. It is also important to mention that rituximab exerts little effect on certain CD20 negative B cells and on mature antibody-producing plasma cells, which may maintain the disease activity despite its presence.

Even though most of the immunotherapies are new to the NMO field, they are widely used in other autoimmune diseases and their side effects are well described in the medical literature. In general, NMO patients tolerate these therapies similarly to other patient populations. However, treatment of NMO patients with long-term immunosuppressants is complicated by their neurological disability and coexistent medical conditions. Therefore, systemic and organ-specific adverse effects should be expected and frequent monitoring is recommended. The most important adverse effects are myelosuppression and secondary leucopenias and lymphopenias. Significant myelosupperssion associated with azathioprine use can occur in patients in whom the critical metabolizing enzyme thiopurine S-methyltransferase (TPMT) is either partially or completely inactivated [28, 53]. The latter can be seen in patients with homozygous or heterozygous mutations in the TPMT gene. These mutations may be found in 10% of the population or associated with intake of enzyme inhibitors such as aspirin, allopurinol, and furosemide. Independently, myelosuppression can also be potentiated by carbamazepine, an anticonvulsant that is commonly used for neuropathic pain [54]. Finally, NMO patients are likely to be treated with multiple immunosuppressive and cytotoxic agents raising concerns about secondary malignancies as well as systemic or opportunistic infections [28, 29, 34, 38].

One should be aware that certain therapeutic agents that are commonly used in MS could actually worsen NMO. In particular, treatment with interferon-beta has been shown to increase disease activity in NMO, as well as to increase anti-aqp-4 antibody titers [55]. It is now recognized that disease mechanisms of MS and NMO involve different T cell subsets. Autoimmunity in MS is driven predominantly by Th1 cells (a T cell subset producing interferon-gamma), whose function is suppressed by interferon-beta. In contrast, NMO is a predominantly Th17-driven disease and administration of interferon-beta potentiates its pro-inflammatory effect on neutrophils and antibody production [56]. In addition, fingolimod and natalizumab may be associated with persisting or worsening NMO activity [57, 58]. Therefore, these medications should be avoided in patients suspected of having NMO. In clinical practice, NMO and NMOSD should be on the differential diagnosis in patients with suspected MS who worsen on interferon-beta, natalizumab, or fingolimod treatment. As a corollary, NMO patients with coexisting systemic autoimmune diseases should be carefully monitored for unintended treatment-induced disease worsening, as more than 30% of all NMO patients may have another autoimmune disease [59].

There is circumstantial evidence of an association between infections and NMO. Disease onset may be preceded by an infectious prodrome in up to 30% of all patients [2]. Chronic infections such as Mycobacterium tuberculosis, human immunodeficiency virus, Helicobacter pylori, and others may be present in patients with NMO [12, 60, 61]. One should consider investigating patients for chronic infections, particularly if environmental or personal risk factors for such infections can be identified. One may also consider the possibility of disease relapses being triggered by more ubiquitous microbial species. These can express immune epitopes with the capacity to cross-react and activate anti-aqp4 specific T and B cells [62–65]. The latter may be relevant to patients with chronic respiratory or bladder problems and decubitus ulcers, who are more prone for infectious complications. Clinical vigilance and at times prophylactic use of antibiotics and changes in immunotherapy may be warranted. In some NMO cases, specific antibiotic (antituberculosis) treatment has been reported to induce disease remission [66]. At this point, there is no available information regarding whether or not vaccination planning should be applied differently to NMO patients. Nonetheless, a clinician should consider a patients' general health status and their current immunosuppressive therapy.

Despite the fact that immunotherapy of NMO takes into account the relative predominance of certain humoral and cellular processes in the disease pathogenesis, it remains nonspecific in nature and produces global immunosuppression. Recently, new experimental approaches directed against more disease-specific immune mechanisms were proposed [67]. Among these is the use of inhibitors of anti-aqp4 antibody binding. An example of this approach is aquaporumab, a nonpathogenic monoclonal antibody against aqp4, which can function as a competitive inhibitor of the disease-associated NMO IgG. This strategy has generated promising results in some of the *in vitro* and *in vivo* models of NMO [68]. Another example is the treatment of patient sera with bacteria-derived endoglycosidase S. Such treatment causes IgG deglycosylation and converts the pathogenic anti-aqp4 antibodies into nonpathogenic target-blocking antibodies [69]. Development and implementation of disease-specific therapeutics may be an important step towards improving the treatment outcomes of the disease and solving some of the clinical dilemmas associated with chronic immunosuppression.

## 5. Conclusion

Clinical and basic science knowledge of NMO has dramatically increased over the last decade. Immunotherapy of NMO is still in its naissance but appears promising and certainly has changed the perception of NMO as an inevitably disabling or fatal disease. Perhaps, the most encouraging aspect is that a large number of treatment options may be used depending on the specific clinical settings. Issues that remain to be addressed include better and earlier recognition of patients with relapsing-remitting disease, identification of prognostic factors of treatment response, development of a biomarker of disease activity, and research on the infectious triggers of the disease. This is complicated by the fact that NMO is a rare disease such that the clinical experience with immunotherapies is still anecdotal. Multicenter, prospective, and controlled studies are required in order to identify the optimal immunotherapies for this disease.
